# Screening of the Candidate Metabolite to Evaluate the Mycelium Physiological Maturation of *Lyophyllum decastes* Based on Metabolome and Transcriptome Analysis

**DOI:** 10.3390/jof10110734

**Published:** 2024-10-23

**Authors:** Lidan Liang, Xizhe Zang, Peijin Zhang, Jingwei Sun, Qingyun Shi, Siyuan Chang, Pengfei Ren, Zhuang Li, Li Meng

**Affiliations:** 1 Shandong Provincial Key Laboratory of Agricultural Microbiology, College of Plant Protection, Shandong Agricultural University, Tai’an 271018, China; 2 State Key Laboratory of Nutrient Use and Management, Shandong Academy of Agricultural Sciences, Jinan 250100, China; 3 Key Laboratory of Wastes Matrix Utilization, Ministry of Agriculture and Rural Affairs, Shandong Academy of Agricultural Sciences, Jinan 250100, China

**Keywords:** edible mushroom, mycelial growth times, yield, secondary metabolism, flavor amino acid, metabolome, transcriptome

## Abstract

*Lyophyllum decastes* is a commercially cultivated rare edible mushroom with high dietary and medicinal value. The mycelium physiological maturation was an important factor to the yield and quality of mushrooms obtained. However, it was impossible to obtain discriminative characteristics represented the maturity level of the mycelia from morphological features. In this article, we screened a candidate metabolite for evaluating the mycelium physiological maturation by metabolomic and transcriptomic analysis during mycelial vegetative growth stages of *L. decastes*. The results showed that mycelial vegetative growth 55 d had reached to physiological maturation according to the yield and quality of *L. decastes*, which the single bottle yield (307 g/bottle), the contents of flavonoids (2.94 mg/g dry weight), and 5 flavor amino acids (glutamate 177.90 μg/g, aspartate 95.74 μg/g, phenylalanine 31.68 μg/g, tyrosine 13.79 μg/g, and alanine 10.99 μg/g) were the highest. In addition, the metabolomic and transcriptomic analysis results showed that biosynthesis of secondary metabolites and amino acid had significant different during mycelial vegetative growth stages, and glutamine may be a candidate indicator to evaluate the mycelial physiological maturation. These findings contribute to improve our knowledge of mycelium physiological maturation and it will be used to increase mushroom yield and quality.

## 1. Introduction

*Lyophyllum decastes* is a highly marketable edible mushroom, owing to its delectable flavor and abundant nutrients [1,2]. Moreover, it has been reported to possess a wide range of health benefits, including antioxidant [3], antidiabetic [4], hypolipidemic [5], immunoregulation [6], and antitumor activity [7]. In recent years, the production of *L. decastes* has increased with the development of industrialized cultivation techniques. China is the largest *L. decastes* producer in the world, with a yield of about 11,590 tonnes in 2020 [8]. However, the current *L. decastes* cultivation techniques cannot ensure stable yield and high quality.

*L. decastes* is a species of macro–fungi of the phylum Basidiomycota [9]. The growth and development of most macro–fungi can be divided into three stages, the vegetative growth stage (mycelia), the transition stage (primordia), and the reproduction growth stage (basidioma) [10]. Compared with other cultivated mushrooms, such as *Hericium erinaceus* and *Flammulina filiformis*, longer mycelial vegetative growth times, are required to reach mycelium physiological maturation for high–quality agronomic traits and yields of *L. decastes*. Elongation of mycelial vegetative growth time is a common growth stage in most edible mushrooms, such as *Lentinula edodes* [11], *Pleurotus eryngii* subsp. *tuoliensis* [12], etc. Notably, *Hypsizigus marmoreus* requires a significant length of time (about 110 to 120 days) from inoculation to harvest [13]. The essence of the mycelium physiological maturation is the nutritional process of mycelial metabolic accumulation, transforming many substances and supplying essential nutrients for basidioma formation. However, longer mycelial growth times do not necessarily indicate higher yield and quality. On the one hand, an excessively long mycelial growth time would yield aged mycelia, with inferior quality and viability. On the other hand, the long cultivation period increases the risk of contamination and severely limits industrialized production. Overall, mycelial growth time requirements vary depending on the species, and extended mycelial vegetative growth time could lead to spawn aging and yield reduction [14]. The vegetative mycelium is mainly involved in nutrient absorption, and nutrient depletion is thought to promote basidioma differentiation. However, the specific role of metabolites in the biological process of the mycelium physiological maturation remains unclear.

Therefore, this study aimed to investigate the effects of different mycelium physiological maturation on yield, the number of primordia, flavonoids and the amino acid contents of *L. decastes*. Moreover, the metabolites and genes of different mycelial growth times were investigated to illustrate their effects during mycelium physiological maturation by widely targeted metabolomic and transcriptomic analysis. The results may present a theoretical reference for selecting mycelial vegetative growth time to achieve high yield and quality mushroom production.

## 2. Materials and Methods

### 2.1. Strain and Cultivation Conditions

The strain of *L. decastes* was obtained from the Shandong Academy of Agricultural Sciences, Shandong, China. It was grown at 25 °C in a potato dextrose agar (PDA) medium for 7 days. Three different mycelial vegetative growth stages 45 d, 55 d, and 65 d were chosen for metabolomic and transcriptomic analyses. Subsequently, the basidioma from the three mycelial growth stages were collected and the amino acid content, flavonoids and yield were detected. The growing medium was sawdust (32%) mixed with 21% corncob, 17% wheat bran, 15% corn flour, 7%soybean peel, 5%cottonseed hulls, and 3% soybean meal based on dry weight, which was adjusted to a moisture content of 65%. The bottles were filled with moistened substrate, and sterilized at 121 °C for 2 h. The cultivation room was maintained at a temperature of 23 °C and 65–75% relative humidity for mycelial colonization. After the substrate was entirely covered with mycelia, the temperature was controlled at 18 °C, and the substrate surface was scratched to stimulate primordia formation. The bottles were opened to allow the development of basidioma under a relative humidity of 95%.

### 2.2. Determination of Flavonoids and Amino Acid Content

Flavonoids content was measured according to our previous study [15]. Contents of amino acids were determined by high performance liquid chromatography (HPLC) analysis. One hundred milligrams of freeze–dried basidioma were suspended in pure water, and the mixture was sonicated for 30 min by ultrasound. Following that, to add 1 mL dichloromethane, and the mixture was centrifuged (13,000 rpm, 5 min, 4 °C) after vortexing for 30 min. Supernatants were collected and filtered by a 0.22 μm membrane. This separation was performed on a C18–Hypersil Gold (Thermo Scientific, Waltham, MA, USA, 3 μm, 100 mm × 2.1 mm) column. Formic acid 0.1% (*v*/*v*) and methanol 100% were used as mobile phases A and B, respectively. In mobile phase B, the chromatographic gradient was set as follows: 0–0.5 min: 4% B; 0.5–2.5 min: from 4% to 35% B; 2.5–4.5 min: from 35% to 77% B; 4.5–6.5 min: from 77% to 100% B; 6.5–6.6 min: from 100% to 4% B; 6.6–10 min, 4% B. There was a total flow rate of 0.2 mL/min, a 3 μL injection volume, and a 40 °C column temperature. The process of mass spectrometry was performed using an ion source called ESI. The ion spray voltage was set at 3500 V, while the sheath gas flow rate was maintained at 35 Arb. Additionally, an auxiliary gas flow rate of 10 Arb was used, and the capillary temperature was set to 325 °C.

### 2.3. Metabolite Extraction

The sample mycelia from different mycelial growth stages were freeze–dried with a Scietz–100F vacuum freeze dryer. A mixer mill (MM 400, Retsch, Düsseldorf, Germany) was used to crush freeze–dried samples with a zirconia bead for 1.5 min at 30 Hz. A solution of 1.2 mL 70% methanol was dissolved in 100 mg of lyophilized powder, vortexed six times for 30 sec each, and stored overnight at 4 °C. For ultra–performance liquid chromatography–tandem mass spectrometry (UPLC–MS/MS) analysis, the extracts were centrifuged for 10 min at 12,000 rpm before being filtered (0.22 μm pore size).

### 2.4. UPLC–MS/MS Conditions

The metabolite extraction was performed using a UPLC–ESI–MS/MS system. 1.8 μm, 2.1 × 100 mm Agilent SB–C18 column was used for separation. In the mobile phase, a mixture of pure water and acetonitrile with 0.1% formic acid and acetonitrile with 0.1% formic acid as solvent A and solvent B, respectively. In this experiment, samples were measured using a gradient program with an initial concentration of 95% A and 5% B, followed by a linear gradient to 5% A and 95% B within nine min, followed by a 1 min maintenance of the composition of 5% A and 95% B. Consequently, within 1.1 min, the composition of 95% A and 5% B was quickly adjusted and maintained for 2.9 min. Flow rate was set at 0.35 mL/min, with a column oven temperature of 40 °C and injection volume of 4 μL. Alternatively, the effluent was sent to a QTRAP–MS system that incorporated an ESI–triple quadrupole. Metabolite analysis was modified from the previous report [16].

### 2.5. Analyses of Qualitative and Quantitative Metabolite

Statistical analysis of metabolite data was performed by log2–transforming and normalizing the data to improve normality. The hierarchical clustering analysis (HCA) and principal component analysis (PCA) of metabolites was performed using R software to examine metabolite accumulation during different accessions. The *p* and fold change values were set to 0.05 and 2.0, respectively. In order to illustrate the number of differential metabolites, Venn diagrams were used. We studied differential metabolites using the Kyoto Encyclopedia of Genes and Genomes (KEGG) database with a *p*–value of 0.01. GraphPad Prism v6.01 was used to visualize all data (GraphPad Software Inc., La Jolla, CA, USA).

### 2.6. RNA Extraction and RNA–Seq

Total RNA was extracted from mycelia samples with mycelial growth times using an RNAprep Pure Plant Kit (DP441, Tiangen, Beijing, China). An Agilent Bioanalyzer 2100 system (Agilent Technologies, Santa Clara, CA, USA) and a NanoPhotometer spectrophotometer (IMPLEN, Westlake Village, CA, USA) were used to quantify the quality of the RNA. mRNA was randomly fragmented after enrichment with Oligo (dT) magnetic beads. First–strand cDNA was synthesized using the M–MuLV reverse transcriptase system. DNA polymerase was used to synthesize second–strand cDNA after RNA strands were degraded by RNase H. A sequencing adapter was ligated to the double–stranded cDNA. Following amplification and purification, cDNA libraries were prepared and sequenced with the Illumina Novaseq6000 system using AMPureXP beads (Beckman Coulter, CA, USA).

### 2.7. Sequence Data Processing

Original image data were converted into sequence reads (raw reads) with the CASAVA base recognition software (version 1.0.0). Fastp was used to remove adapters and poor–quality sequences with more than 50% Qphred ≤ 20 bases. In order to measure the base quality, we calculated the GC content of clean reads and then used FastQC (version 1.0.0) to calculate Q20 and Q30 values. In addition, the RNA–seq data were assembled and analyzed as no reference genome sequences. Fragments per Kilobase of transcript per Million fragments mapped (FPKM) was used to determine gene expression levels. We defined differentially expressed genes (DEGs) as those whose expression was changed by log2fold in at least one direction and has a false discovery rate of 0.05 or less. Based on Nr, Pfam, Swiss–Prot, KEGG, and GO databases, gene functions were annotated.

### 2.8. Quantitative Real–Time PCR

The total RNA was extracted using RNAiso Plus Kit (Takara, Kusatsu, Japan). First–strand cDNA was synthesized using EasyScript One–Step gDNA removal and cDNA synthesis superMix (TransGen, Beijing, China). The transcriptional levels of genes in different mycelial growth stages were analyzed using the LightCycler 96 SW 1.1 instrument and SYBR Green (TransGen, Beijing, China) for quantitative real–time PCR.

Gene expression data from different samples was normalized using *UBC* as an internal reference gene for accurate and reliable gene expression analysis. The cycle threshold (Ct) of each target gene was compared with the relative expression of the corresponding internal reference gene using the 2^−ΔΔCt^ method. The primers used were shown in Table 1.

### 2.9. Detection of Glutamine Content

Glutamine content detection kit was supplied by Shanghai Jing Key Biotechnology Co., Ltd. (Shanghai, China) Glutamine content was determined based on the following reaction: Glutamine → Glutamate + NAD → 2–Oxoglutrate + NADH + NH_4_^+^, NADH + WST → Formazan. Then the formazan was measured at 450 nm by a spectrophotometer. The glutamine content was calculated as: Glutamine content (μmol/g) = ∆A (sample) × 0.4 ÷ ∆A (standard) − W, where ∆A was the average absorbance difference per minute, W was the mass of the sample, and the standard solution was 0.4 μmol/mL glutamine.

## 3. Results

### 3.1. The Yield and Quality of L. decastes

**Figure 1:**
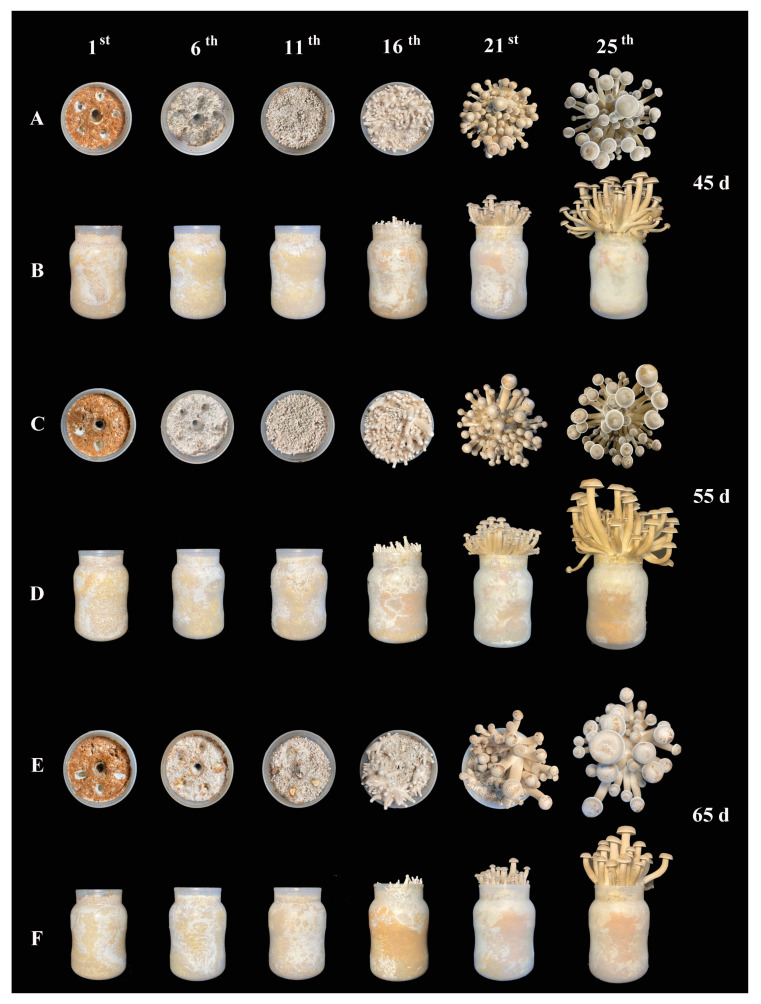
The conditions of basidioma development in different mycelial vegetative growth stages. (**A**,**B**) were the basidioma development in different mycelial vegetative growth stage of 45 d. (**C**,**D**) were the basidioma development in different mycelial vegetative growth stage of 55 d. (**E**,**F**) were the basidioma development in different mycelial vegetative growth stage of 65 d. 1st, 6th, 11th, 16th, 21st, 25th were the first, sixth, eleventh, sixteenth, twenty–first and twenty–fifth day after substrate surfaces were scratched, respectively.

**Figure 2:**
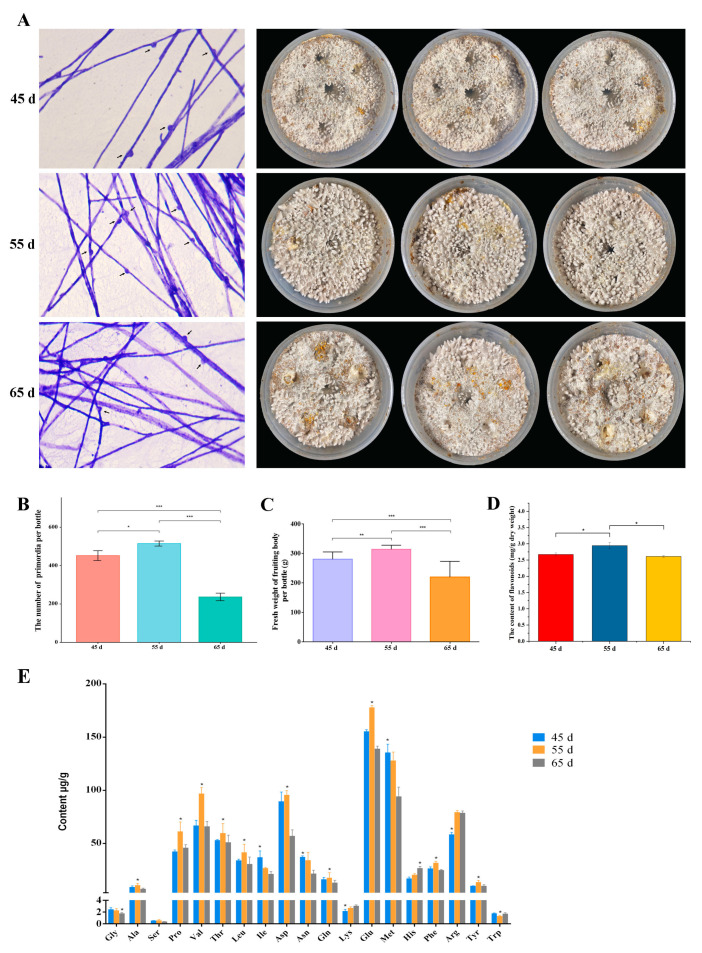
The yield, flavonoids and amino acid content in different mycelial vegetative growth stages. (**A**) Clamp connection and primordia. Clamp connection indicated by black arrows. (**B**) The number of primordia. (**C**) Fresh weight of basidioma. (**D**) The content of flavonoids. (**E**) The content of amino acid. Statistical significance was determined using Students t test (one asterisk, *p* < 0.05; two asterisks, *p* < 0.01; three asterisks, *p* < 0.001). Gly, Glycine; Ala, Alanine; Ser, Serine; Pro, Proline; Val, Valine; Thr, Threonine; Leu, Leucine; Ile, Isoleucine; Asp, Aspartic acid; Asn, Asparagine; Gln, Glutamine; Lys, Lysine; Glu, Glutamic acid; Met, Methionine; His, Histidine; Phe, Phenylalanine; Arg, Arginine; Tyr, Tyrosine; Trp, Tryptophane.

The influence of different mycelial vegetative growth stages (45 d, 55 d, and 65 d) on the yield and quality of *L. decastes* was investigated, observing the development of primordia and basidioma (Figure 1). Based on the statistical analysis of thirty bottles at each mycelial growth stage, the highest growth potential of basidioma was achieved in the 55 d group. Microscopic observations of fungal hyphae in the three groups showed that the density of hyphae in the 45 d was sparse, and clamp connection also was slightly fewer than 55 d group. Furthermore, fewer the clamp connection was observed in 65 d group in the case of similar hyphal density compared with 55 d group (Figure 2A). An average of 515 emerged primordia was observed in the 55 d group, which was significantly higher than in the 45 d (452 on average) and 65 d (237 on average) groups (Figure 2B). Normally, higher numbers of primordia indicate a higher yield in mushroom production [17].

Furthermore, the yield, flavonoids and amino acid content of basidioma were detected in different mycelial vegetative growth stages. The results revealed that the single bottle yield at 55 d was the highest (307 g/bottle), which was significantly higher than that in the 45 d (280.33 g/bottle) and 65 d (219.90 g/bottle) groups (Figure 2C). The content of flavonoids was higher (2.94 mg/g dry weight) in 55 d basidioma than that in 45 d (2.67 mg/g dry weight) and 65 d (2.61 mg/g dry weight) groups (Figure 2D). Among the 20 kinds of free amino acids, every amino acid except cysteine was detected by HPLC in all fruiting bodies. Interestingly, 5 flavor amino acids (alanine, aspartic acid, glutamic acid, phenylalanine, and tyrosine) showed significantly higher levels in the basidioma of the 55 d group compared to the 45 d and 65 d (Figure 2E) groups. The glutamic acid content was the highest (177.90 μg/g dry weight) in the 55 d basidioma, followed by aspartic acid (95.74 μg/g dry weight), phenylalanine (31.68 μg/g dry weight), tyrosine (13.79 μg/g dry weight) and alanine (10.99 μg/g dry weight). These results revealed that mycelial vegetative growth in 55 d of *L. decastes* had the highest yield, flavonoids and flavor amino acid content in basidioma.

### 3.2. The Metabolome Profiling of L. decastes

**Figure 3:**
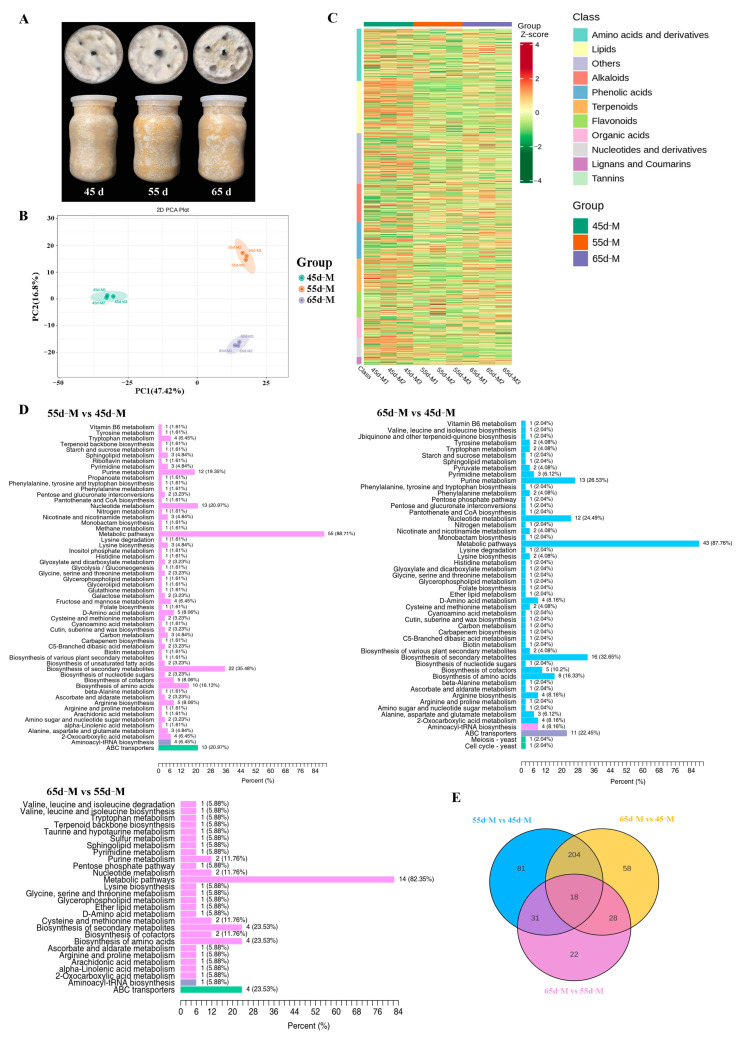
Widely Targeted Metabolomic Analysis. (**A**) Samples were subjected to metabolomics analysis. (**B**) Principal component analysis (PCA) across all metabolites and samples. (**C**) Heat map of identified metabolites. (**D**) KEGG pathway classification diagram. (**E**) Venn diagram of differences among the groups.

To better understand the effects of different mycelial vegetative growth stages (45 d, 55 d, and 65 d) on the metabolites, a widely targeted metabolomic analysis was performed. The samples from various mycelial vegetative growth stages were analyzed by using the UPLC–MS/MS detection platform (Figure 3A). Principal component analysis (PCA) was performed to assess the quality of samples, showing high consistency between biological replicates, while each group was completely separated. PC1 and PC2 showed variances of 47.42% and 16.8%, respectively (Figure 3B).

A total of 1214 metabolites were detected, including amino acids and derivatives, lipids, and others (Figure 3C). A differentially accumulated metabolite (DAM) is one with a fold change of ≥2, a fold change of ≤0.5, and a VIP of ≥1. Based on these criteria, a total of 334 (135 upregulated and 199 downregulated), 308 (142 upregulated and 166 downregulated), and 99 (68 upregulated and 31 downregulated) DAMs were detected in the 55 d vs. 45d, 65 d vs. 45 d, and 65 d vs. 55 d comparisons, respectively (Table S1). There was a greater number of DAM was found in 55 d vs. 45d, implying that the magnitude of the metabolite changes occurred at 55 d.

Moreover, KEGG was used to annotate the DAMs. According to KEGG, these DAMs were mostly involved in metabolic pathways (ko01100), secondary metabolite biosynthesis (ko01110), nucleotide metabolism (ko01232), purine metabolism (ko00230), and amino acid biosynthesis (ko01230) (Figure 3D). The Venn diagram results showed 81, 58, and 22 unique metabolites in the 55 d vs. 45 d, 65 d vs. 45 d, and 65 d vs. 55 d comparisons, respectively (Figure 3E, Table S2). Among the 81 unique metabolites between 55 d vs. 45 d, 31% were secondary metabolites (flavonoids, terpenoids, etc.), 19% were lipids, 14% were amino acids and derivatives, and 11% were alkaloids. The 58 unique metabolites between 65 d vs. 45 d comprised 40% secondary metabolites, 12% lipids, 12% phenolic acids, and 10% amino acids and derivatives. There were 36% secondary metabolites, 32% amino acids and derivatives among the 22 unique metabolites of 65 d vs. 55 d. Furthermore, 18 DAMs (56% secondary metabolites, 11% amino acids and derivatives, and others) were found in all three groups. As a result of these findings, DAMs involved in secondary metabolism and amino acid biosynthesis appear to play a significant role in mycelium physiological maturation.

### 3.3. The Transcriptome Profiling of L. decastes

**Figure 4:**
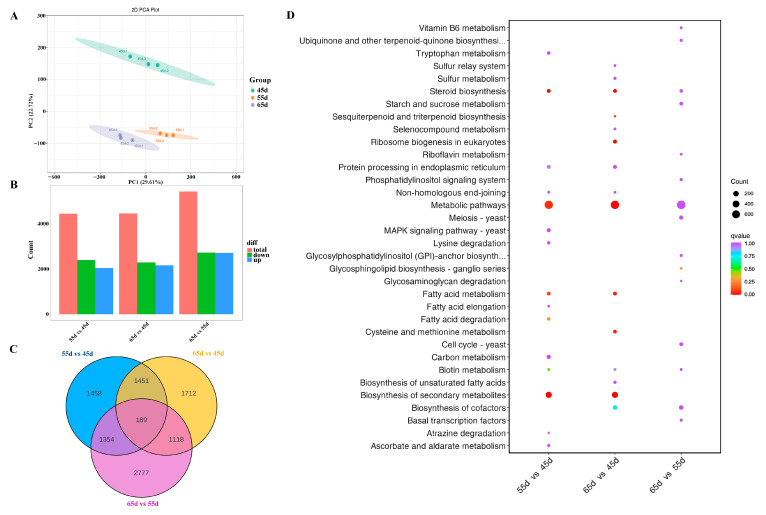
RNA–Seq Analysis. (**A**) Principal component analysis (PCA) across all genes and samples. (**B**) The count of differentially expressed genes. (**C**) Venn diagram of differentially expressed genes among the groups. (**D**) KEGG function classification of differentially expressed genes.

Transcriptome raw reads have been deposited in NCBI’s BioProject, accession number PRJNA983922. Nine high–quality RNA libraries were constructed, resulting in 92.1 GB of high–quality clean reads, 97.8% Q20 and 93.92% Q30. A PCA analysis was performed on the RNA–Seq data set of 9 samples (Figure 4A). As with the metabolome, the PCA on RNA–Seq showed similar patterns, with groups for different mycelial vegetative growth stages clearly defined, suggesting similar gene expression between samples within each category.

There were 4452 (2044 upregulated and 2408 downregulated), 4470 (2171 upregulated and 2299 downregulated) and 5438 (2712 upregulated and 2726 downregulated) differentially expressed genes (DEGs) in the 55 d vs. 45 d, 65 d vs. 45 d and 65 d vs. 55 d comparisons (Figure 4B, Table S3). The Venn results showed that there were 1458, 1712, and 2777 unique DEGs in the 55 d vs. 45 d, 65 d vs. 45 d, and 65 d vs. 55 d comparisons, respectively (Figure 4C). Moreover, 189 DEGs were found in all three groups. KEGG pathway enrichment analysis results illustrated that the DEGs were associated with metabolic pathways (Figure 4D). The current results were consistent with those obtained in the metabolomic study (Figure 3D).

### 3.4. Biosynthesis of Secondary Metabolites in L. decastes

**Figure 5:**
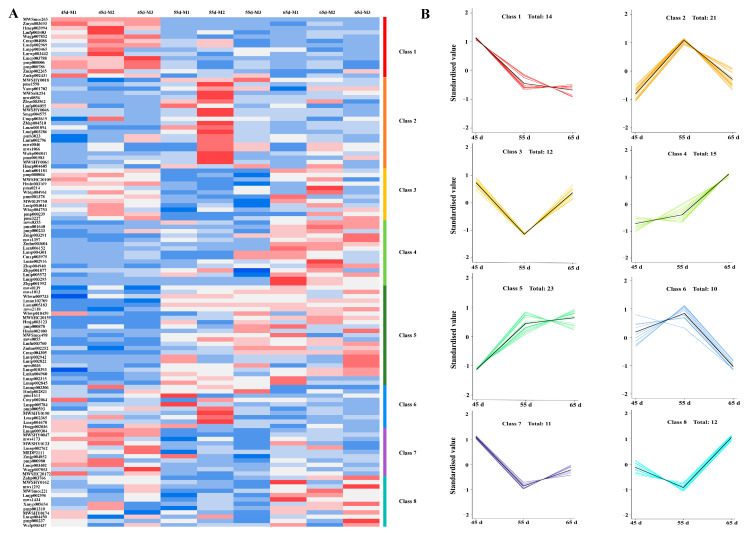
The flavonoids metabolites in different mycelial vegetative growth stages. (**A**) The accumulation of 181 metabolites; (**B**) k–means analysis.

To further analyze the dynamic changes in secondary metabolites biosynthesis in different mycelial vegetative growth stages of *L. decastes*, the DAMs identified secondary metabolites were explored. 181 metabolites, including flavonoids, lignans and coumarins, were identified in different mycelial growth stages (Figure 5A, Table S4). This enriched data set was segregated into eight classes based on similarity of expression profile across the data set using a k–means clustering algorithm (Figure 5B). The result showed that a total of 41 metabolites with differentially accumulated in at least one stage. There were 26 DAMs (15 upregulated and 11 downregulated) in 55 d vs. 45 d, and 27 DAMs (19 upregulated and 8 downregulated) in 65 d vs. 45 d. There were 12 DAMs (10 upregulated and 2 downregulated) in 65 d vs. 55 d. These results implied that the secondary metabolites were gradually increased with the mycelium physiological maturation.

**Figure 6:**
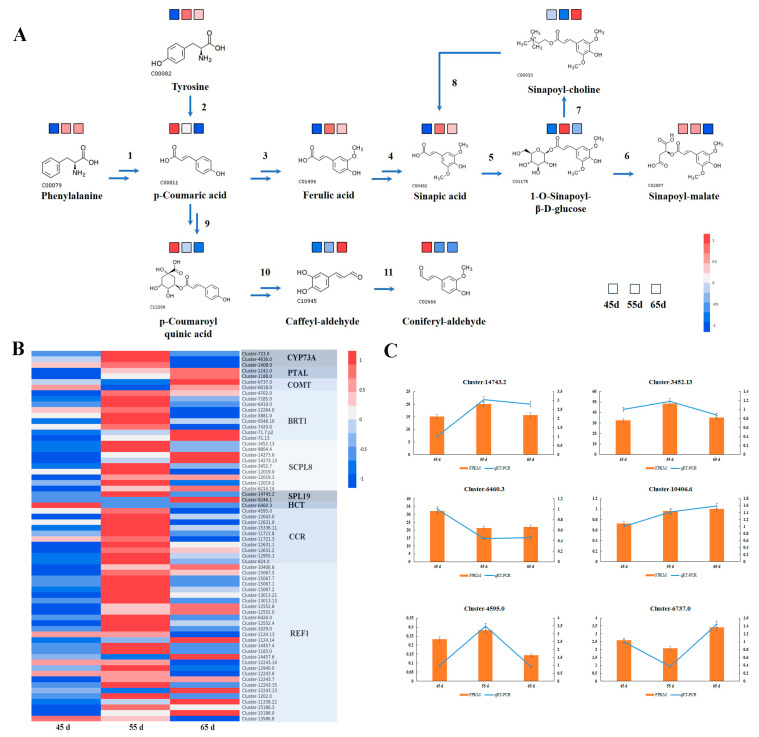
Phenylpropanoid biosynthesis in *Lyophyllum decastes* (**A**) The phenylpropanoid biosynthesis pathway. 1: trans–cinnamate 4–monooxygenase, CYP73A; 2: phenylalanine/tyrosine ammonia–lyase, PTAL; 3, 4: caffeic acid 3–O–methyltransferase/acetylserotonin O–methyltransferase, COMT; 5: sinapate 1–glucosyltransferase, BRT1; 6: serine carboxypeptidase–like 8, SCPL8/SNG1; 7: serine carboxypeptidase–like 19, SCPL19/SNG2; 8: sinapine esterase, SE; 9: shikimate O–hydroxycinnamoyltransferase, HCT; 10: cinnamoyl–CoA reductase, CCR; 11: coniferyl–aldehyde dehydrogenase, REF1. (**B**) The heatmap of genes encoding key enzymes. (**C**) The gene expression level. FPKM, fragments per kilobase of transcript per million fragments mapped, it’s represented by the principal axis. qRT–PCR, quantitative real–time PCR, it’s represented by the secondary axis.

To obtain insight into the major metabolic pathway of secondary metabolites, phenylpropanoid biosynthesis was investigated (Figure 6A). The results revealed that tyrosine and phenylalanine exhibited similar tendencies, they started accumulated in 55 d. Then, the conversions of tyrosine and phenylalanine into p–coumaric acid, and subsequently into ferulic acid and p–coumaroyl quinic acid, which were eventually converted to sinapoyl–malate and coniferyl–aldehyde, respectively. The results showed that p–coumaric acid and p–coumaroyl quinic acid were continuously decreasing with increasing mycelial growth time. The accumulated ferulic acid, sinapic acid, 1–o–sinapoyl–β–D–glucose, and sinapoyl–malate were noted to have the highest accumulation in 55 d. The caffeyl–aldehyde was increasing with mycelial growth time, whereas coniferyl–aldehyde showed the opposite trend. The expressions of the key genes controlling phenylpropanoid biosynthesis in the different mycelial vegetative growth times were measured. The transcriptome data clearly showed that the expressions of key genes, CYP73A, BRT1, CCR, and most of genes encoding REF1, SCPL8 were higher in 55 d group than that in 45 d and 65 d groups (Figure 6B). The expression of PTAL was increased with the mycelial growth times, whereas the expression of COMT, HCT were the lowest in 55 d group. One of two encoding SCPL19 gene expression was the highest in 55 d group, another gene expression was increased with the mycelial growth times. Partial quantitative real–time PCR results were consistent with transcriptomic results (Figure 6C). Collectively, the results indicated a higher amount of metabolite and gene expression levels in 55 d, consistent with the trend in total flavonoids content (Figure 2D).

### 3.5. Biosynthesis of Amino Acids in L. decastes

To gain further insight into the effects of amino acid biosynthesis in different mycelial vegetative growth stages of *L. decastes*, the DAMs in this pathway were explored. There were 10 DAMs in the biosynthesis amino acids of 55 d vs. 45 d, including 8 upregulated metabolites (Glycerone–P, Cystathionine, Aspartate, Lysine, Glutamine, L–Argininosuccinate, N–Acetyl–ornithine, and Proline) and 2 downregulated metabolites (LL–2,6–Diaminopimelate and N–Acetyl–L–glutamate) (Figure S1A). The results showed that the metabolite characteristics in the biosynthesis of amino acids were basically similar in 55 d vs. 45 d and 65 d vs. 45 d. There were 8 DAMs between the 65 d and 45 d groups, including 6 metabolites with the same expression patterns as in 55 d vs. 45 d, and 2 downregulated metabolites (alpha–Isopropylmalate and S–Adenosylhomocysteine) (Figure S1B). However, only 4 DAMs were found in 65 d vs. 55 d, including 2 upregulated metabolites (Leucine and LL–2,6–Diaminopimelate) and 2 downregulated metabolites (S–Adenosylhomocysteine and Cystathionine) (Figure S1C).

Furthermore, 148, 173, and 38 genes were associated with amino acid biosynthesis in the 55 d vs. 45 d, 65 d vs. 45 d, and 65 d vs. 55 d comparisons, respectively. These results showed that more genes were involved in the biosynthesis of amino acids as the mycelial growth time was extended. However, the 65 d vs. 55 d comparison showed a slightly higher number of amino acid biosynthesis–related genes, implying that 55 d was a tipping point of mycelium physiological maturation in *L. decastes*.

**Figure 7:**
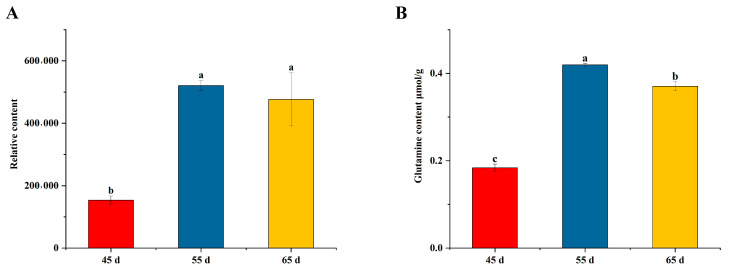
The glutamine content in different mycelial vegetative growth stages. (**A**) Metabolic data; (**B**) The spectrophotometer measurement. Different superscript lowercase letter (a, b and c) indicated significant difference (*p* < 0.05).

Notably, glutamine may be a potential indicator for mycelium physiological maturation. The differential metabolite analysis showed that glutamine was significantly increased in the 55 d group (Figure 7A). In particular, no significant difference in glutamine was found in the differential metabolite analysis between the 55 d and 65 d groups. The result measured using a spectrophotometer was consistent with the findings in metabolomic analysis (Figure 7B). Moreover, the glutamine content of basidioma originated from the 55 d group was the highest (Figure 2E). It was also significant hub metabolite in the amino acids and phenylpropanoid biosynthesis. Glutamine not only participated in the synthesis of various amino acids but was also involved in numerous physiological processes [18], including metabolic pathways [19], biosynthesis of secondary metabolites [20], nucleotide metabolism [21], etc. Therefore, these findings indicated that glutamine may be a candidate indicator to evaluate mycelial physiological maturation.

## 4. Discussion

For mushroom crops, precise determination optimum mycelial vegetative growth time has been a challenging aspect of their production and quality. In this study, some major differential accumulation metabolites and expression genes were identified through combined metabolomic and transcriptomic analysis. By comparative metabolomics analysis, we investigated the mycelial growth time and their roles in the accumulation of secondary metabolites and amino acid synthesis in *L. decastes*. This approach provides a new evaluation method to the mycelium physiological maturation.

In the current study, transcriptome analysis has been the most utilized approach to study basidioma development at the molecular level, such as *Ganoderma lucidum* [22,23], *Flammulina filiformis* [24], *Lentinula edodes* [25], *Dictyophora indusiate* [26], etc. These studies focused on the differences among the mycelia, primordia, and basidioma stages. These findings provide meaningful information about the growth and development of fungi. However, the changing profile during the mycelial vegetative growth stage was overlooked.

*H. marmoreus* is an important edible mushroom species in Asia, previously reported that the mycelium of *H. marmoreus* requires 110 days of growth to ensure high quality and yield [13]. The 90–day sample had a shorter stipe (by 1 cm) and lower yield (by 59 g) than the 110–day sample. The longer the mycelial vegetative growth time, the better the yield in *H. marmoreus*. However, this study found that mycelial vegetative growth stage 55 d in *L. decastes* had the highest yield, whereas extended mycelial growth time decreased the yield. Therefore, the optimal mycelial vegetative growth time varies significantly between mushrooms, and longer mycelial growth times are not always favorable.

In a comparable study, it reported the roles of amino acid metabolism in basidioma formation at different ripening times (30, 50, 70, 90, and 100 d) in *H. marmoreus* [13]. The results showed that the different gene expressions of primordia (collected from five ripening times) were mainly associated with amino acid metabolism, which may be key factors affecting mycelium physiological maturation. Similarly, the amino acids and derivatives also showed significant changes in the different mycelial vegetative growth stages in *L. decastes*. Amino acids are the basic substances that constitute proteins, maintain normal metabolism, and provide a material basis for life activities [27]. Collectively, these data indicated that amino acid biosynthesis and metabolism play an important role during mycelium physiological maturation in most mushrooms.

Previous studies reported that the identified candidate genes related to the development of bailinggu (*P. tuoliensis*), including Stage I (undifferentiated mycelia of the physiological after–ripening stage), Stage II (cold stimulation of Stage I mycelia), Stage III (primordia), and Stage IV (basidioma) [28]. The results showed that the number of unigenes was associated with the cellular process and metabolic process, implying that the metabolic process changed significantly during development, which was in accordance with the results of our study.

According to a previous study, few metabolites were identified as potential indicators of physiological maturation in *P. tuoliensis* mycelia by metabolomic analysis [29]. The pyrimidine synthesis pathway was found to be significantly activated during mycelium physiological maturation of *P. tuoliensis*. Furthermore, they perceived that N–carbamoyl–L–aspartate (CA–asp) could be a potential indicator for the physiological maturation of mycelia, as it was significantly increased on day 35, during the mycelium physiological maturation period. However, in our study, only 1–3 metabolites (4.84–6.12%) of the pyrimidine metabolism were differentially accumulated during various mycelial vegetative growth stages of *L. decastes*. This divergence may be greatly influenced by species and environmental factors, including mycelial culture period, temperature, and so on.

This is the first study to investigate the yield, flavonoids, amino acid content, biosynthesis of secondary metabolite and amino acids in different mycelial vegetative growth stages of *L. decastes*. The findings provide important information on the effects of different mycelium physiological maturation to improve the yield and quality of mushroom production.

## 5. Conclusions

This study elucidated the effects of different mycelial vegetative growth times on amino acid and phenylpropanoid biosynthesis based on metabolomics and transcriptomics data. The results showed that mycelial vegetative growth stage 55 d provided the optimal yield and basidioma amino acid content in *L. decastes*. Notably, the findings of this study indicated that glutamine may be a candidate indicator to evaluate mycelial physiological maturation. These results implied that the biosynthesis of amino acids play an important role in the mycelium physiological maturation, thus influencing yield and quality.

## Figures and Tables

**Table 1:** List of primer sequences.

Primer	Sequence 5′–3′
Lyd–UBC–qPCR–F	GAGAAGGCACTTCACGGTCA
Lyd–UBC–qPCR–R	CCAACGTCTCGAACACTGGA
Lyd–Cluster–6737.0–qPCR–F	ATATGTTTGCCAGCGTCCCA
Lyd–Cluster–6737.0–qPCR–R	AAACGTCTTGGGCCCCTATG
Lyd–Cluster–3452.13–qPCR–F	ATCCCGACACAGCTCCTTTG
Lyd–Cluster–3452.13–qPCR–R	CTACTCCGACGGGTTGATCG
Lyd–Cluster–14743.2–qPCR–F	CAAGCCGCTGATCCGAAATG
Lyd–Cluster–14743.2–qPCR–R	CCCAAGGGGCTCTCAAGAAG
Lyd–Cluster–6460.3–qPCR–F	GATTCATCCGCGTTGTCTGC
Lyd–Cluster–6460.3–qPCR–R	TCAACGTCACGAGTTCCCAG
Lyd–Cluster–4595.0–qPCR–F	GGAGATTGAACCGGACAGCA
Lyd–Cluster–4595.0–qPCR–R	TGCTGAGAAGAAGGCATGGG
Lyd–Cluster–10406.6–qPCR–F	TGACCCACTCGTCGTTTCAG
Lyd–Cluster–10406.6–qPCR–R	CCAGTCTGCTTCTGCAGTCA

## Data Availability

Any data or material that support the findings of this study can be made available by the corresponding author upon request.

## Supplementary Materials

The following supporting information can be downloaded at: https://www.mdpi.com/article/10.3390/jof10110734/s1, Table S1. The differentially accumulated metabolites in different mycelial vegetative growth stages; Table S2. Venn results in different mycelial vegetative growth stages; Table S3. The differentially expressed genes in different mycelial vegetative growth stages; Table S4. The flavonoids, lignans and coumarins in different mycelial vegetative growth stages; Figure S1 Biosynthesis of amino acids.
