# Supplementary material for: Screening of the Candidate Metabolite to Evaluate the Mycelium Physiological Maturation of Lyophyllum decastes Based on Metabolome and Transcriptome Analysis
Source: J Fungi (Basel). 2024 Oct 23;10(11):734. doi: 10.3390/jof10110734 (PMC11596021; doi:10.3390/jof10110734)
Supplement: Supplementary file 1 [file jof-10-00734-s001.zip › jof-3233970-supplementary/Supplementary Materials.docx]

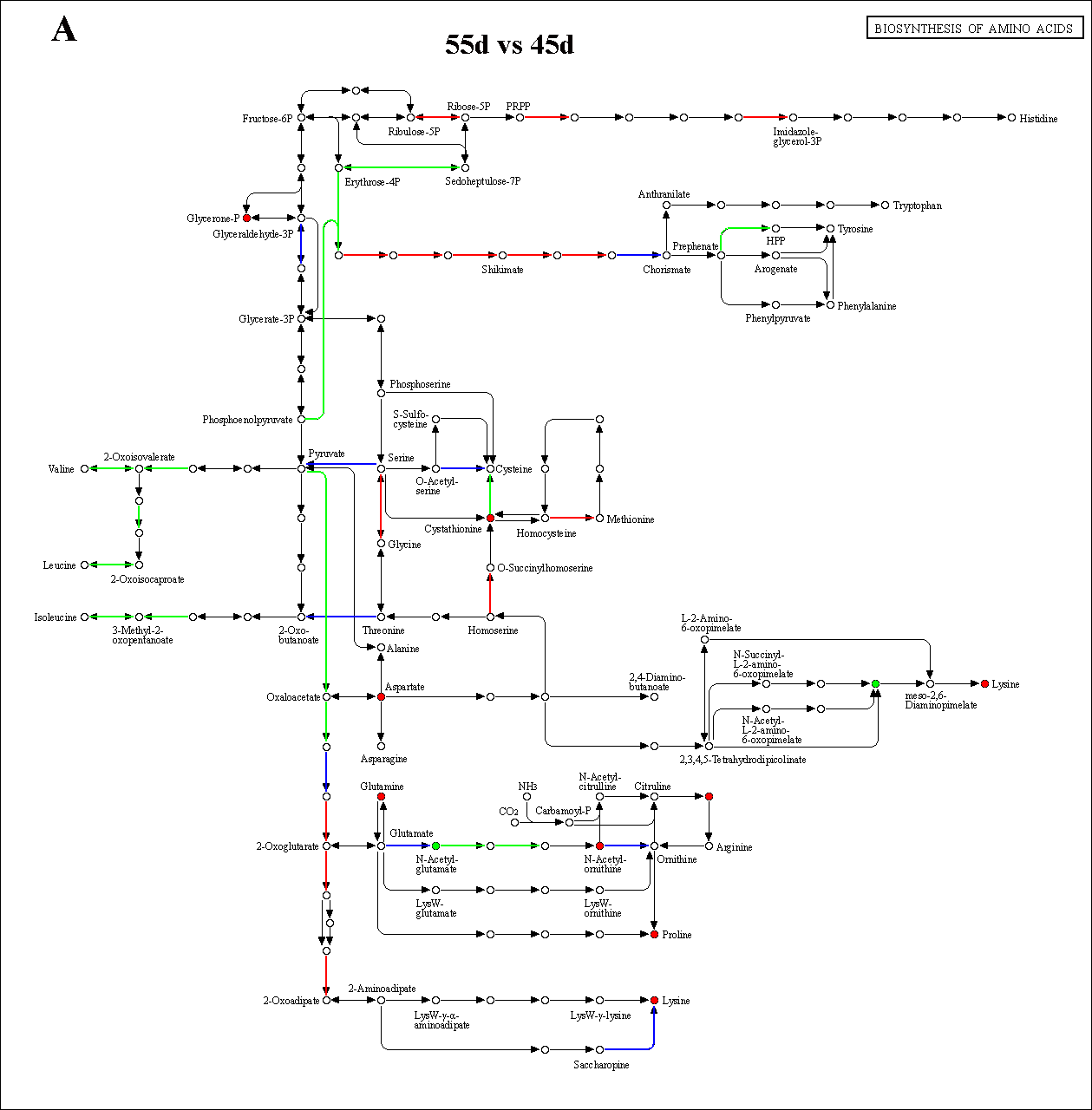


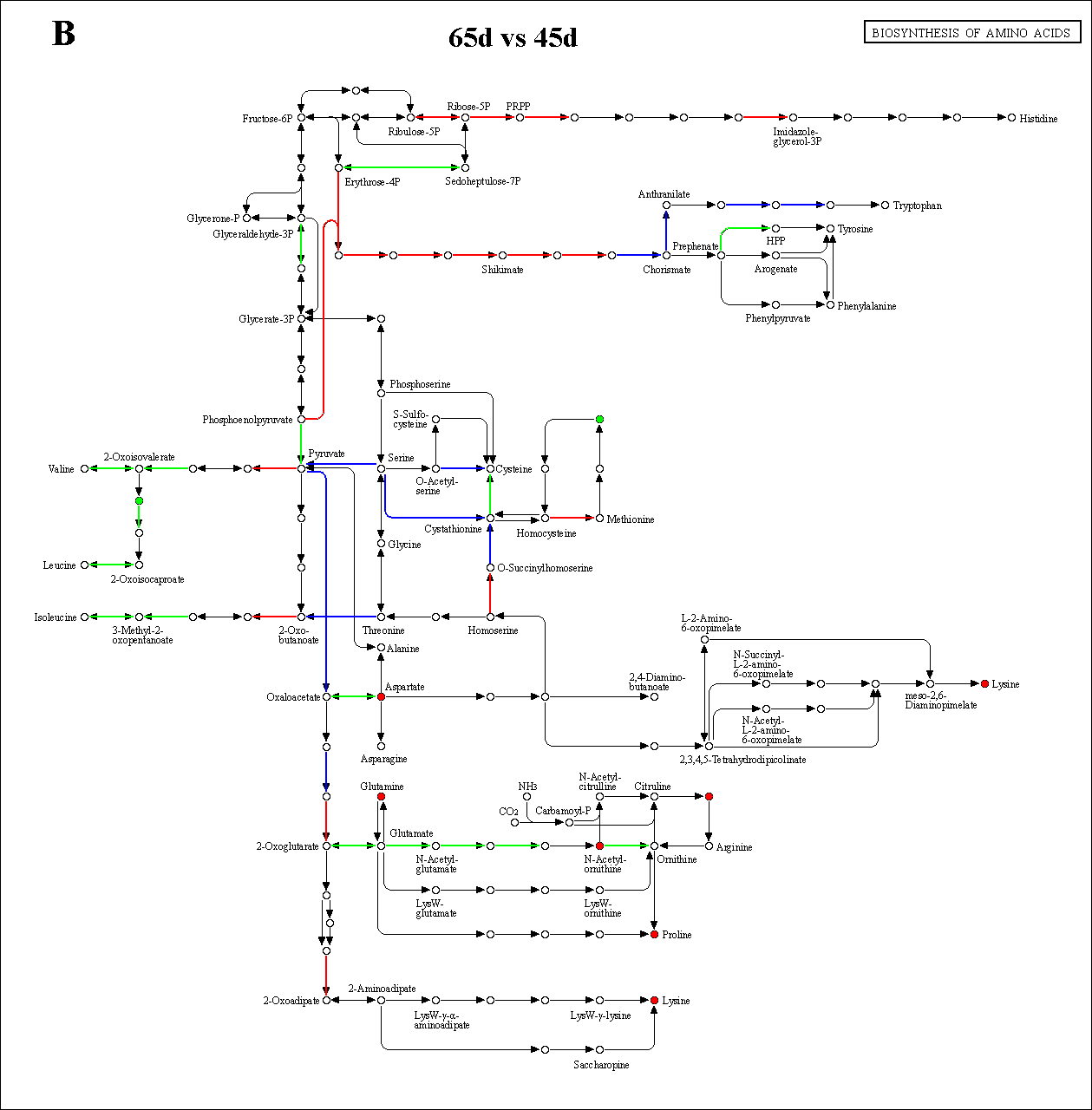


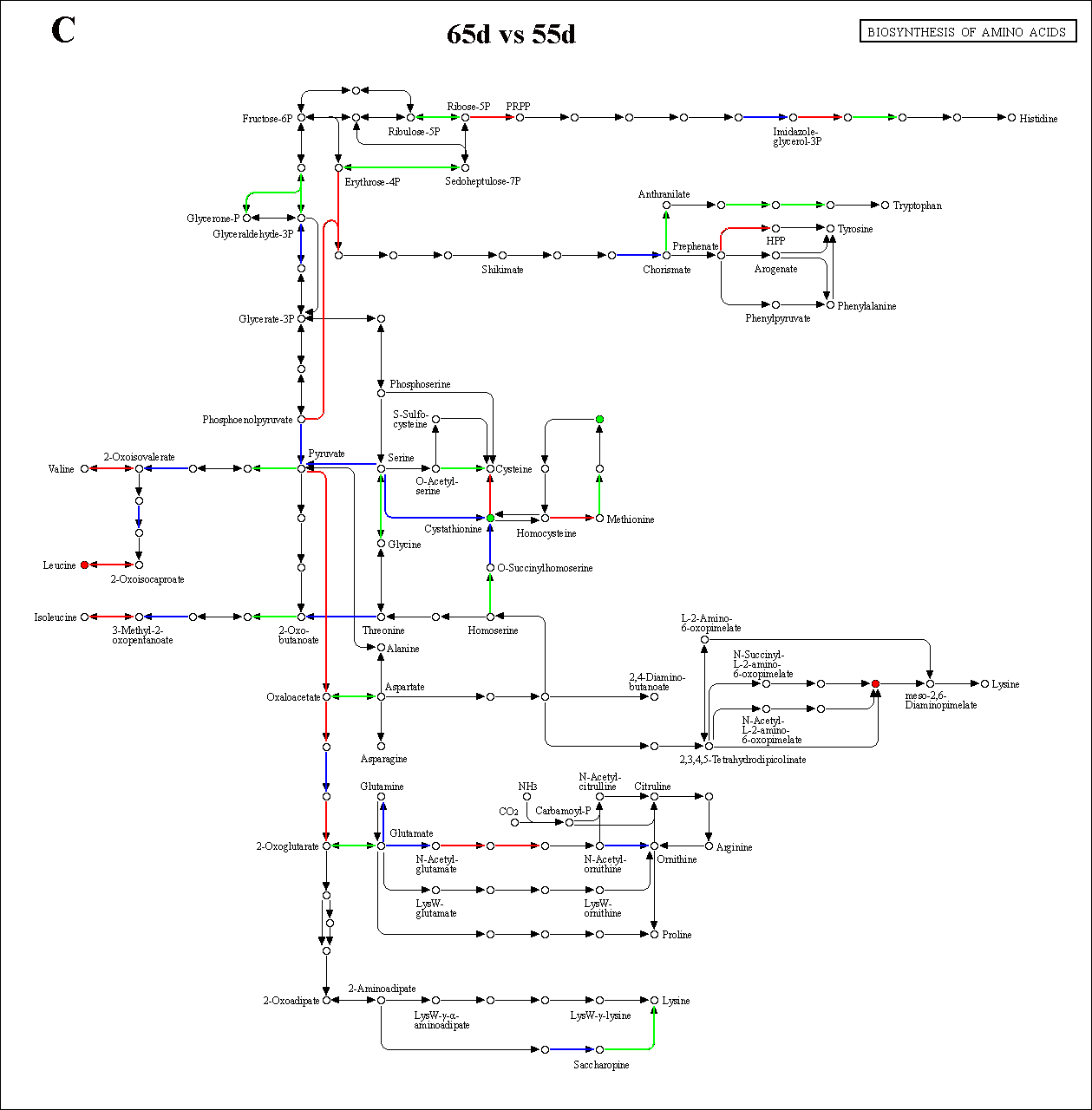


***Figure S1.*** Biosynthesis of amino acids*.*

The circles and arrows represent metabolites and genes, respectively; red: up-regulated, blue: both up-regulated and down-regulated; green: down-regulated.
