# Supplementary material for: Screening of the Candidate Metabolite to Evaluate the Mycelium Physiological Maturation of Lyophyllum decastes Based on Metabolome and Transcriptome Analysis
Source: J Fungi (Basel). 2024 Oct 23;10(11):734. doi: 10.3390/jof10110734 (PMC11596021; doi:10.3390/jof10110734)
Supplement: Supplementary file 1 [file jof-10-00734-s001.zip › jof-3233970-supplementary/Supplementary Materials.pdf]

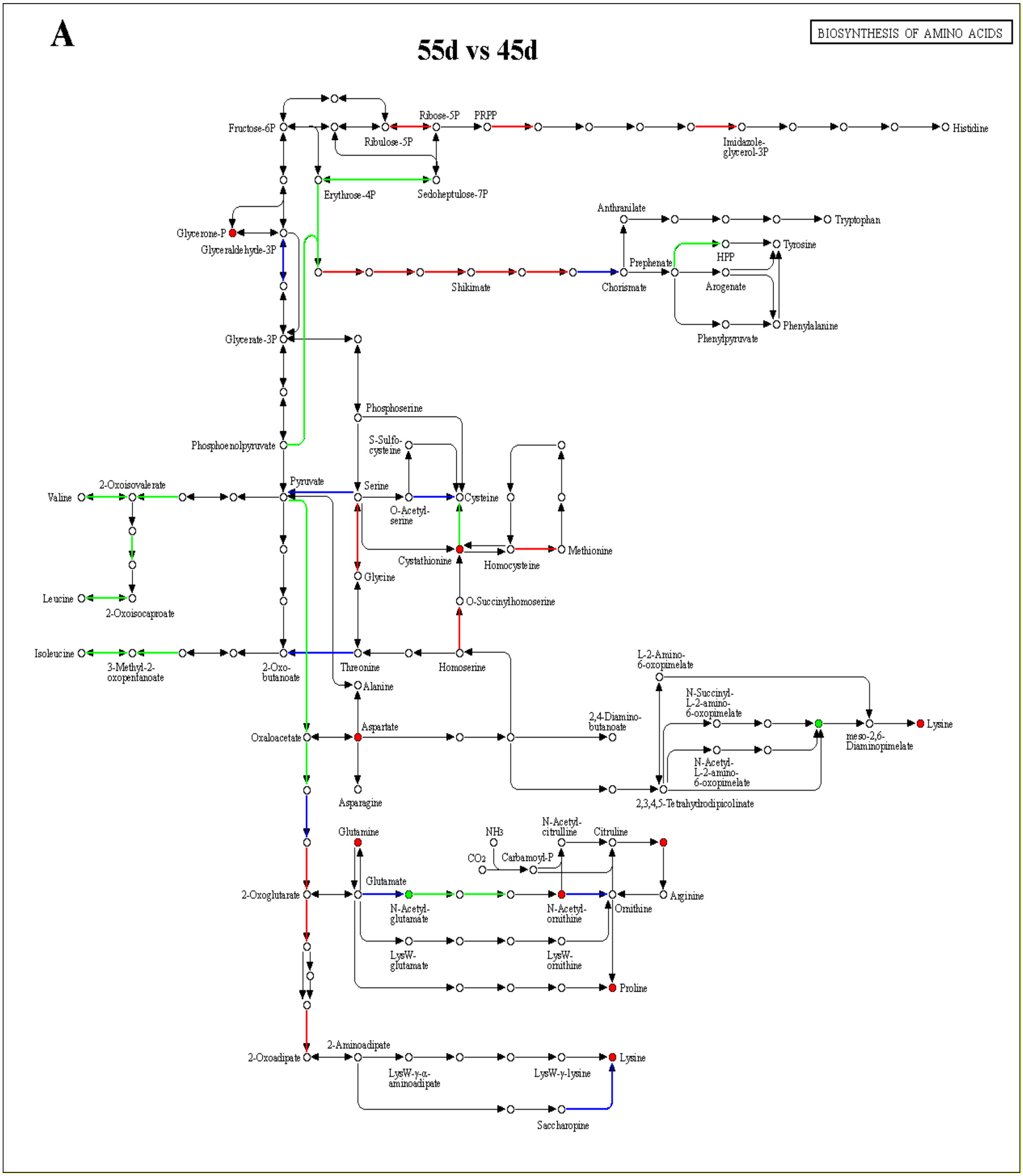
A
55d vs 45d
BIOSYNTHESIS OF AMINO ACIDS
Fructose-6P
Ribose-5P
PRPP
Ribulose-5P
Imidazole-glycerol-3P
Histidine
Erythrose-4P
Sedoheptulose-7P
Anthranilate
Tryptophan
Tyrosine
HPP
Prephenate
Glycerone-P
Glyceraldehyde-3P
Shikimate
Chorismate
Arogenate
Phenylpyruvate
Phenylalanine
Glycerate-3P
Phosphoserine
Phosphoenolpyruvate
S-Sulfo-cysteine
Pyruvate
Serine
Cysteine
2-Oxoisovalerate
Valine
O-Acetyl-serine
Cystathionine
Homocysteine
Methionine
Glycine
Leucine
2-Oxoisocaproate
O-Succinylhomoserine
Isoleucine
3-Methyl-2-oxopentanoate
2-Oxo-butanoate
Threonine
Homoserine
L-2-Amino-6-oxopimelate
Alanine
N-Succinyl-L-2-amino-6-oxopimelate
Aspartate
2,4-Diamino-butanoate
Oxaloacetate
meso-2,6-Diaminopimelate
Lysine
N-Acetyl-L-2-amino-6-oxopimelate
Asparagine
2,3,4,5-Tetrahydrodipicolinate
Glutamine
NH3
N-Acetyl-citrulline
Citruline
CO2
Carbamoyl-P
Glutamate
2-Oxoglutarate
Arginine
N-Acetyl-glutamate
N-Acetyl-ornithine
Ornithine
LysW-glutamate
LysW-ornithine
Proline
2-Aminoadipate
2-Oxoadipate
Lysine
LysW-γ-α-aminoadipate
LysW-γ-lysine
Saccharopine

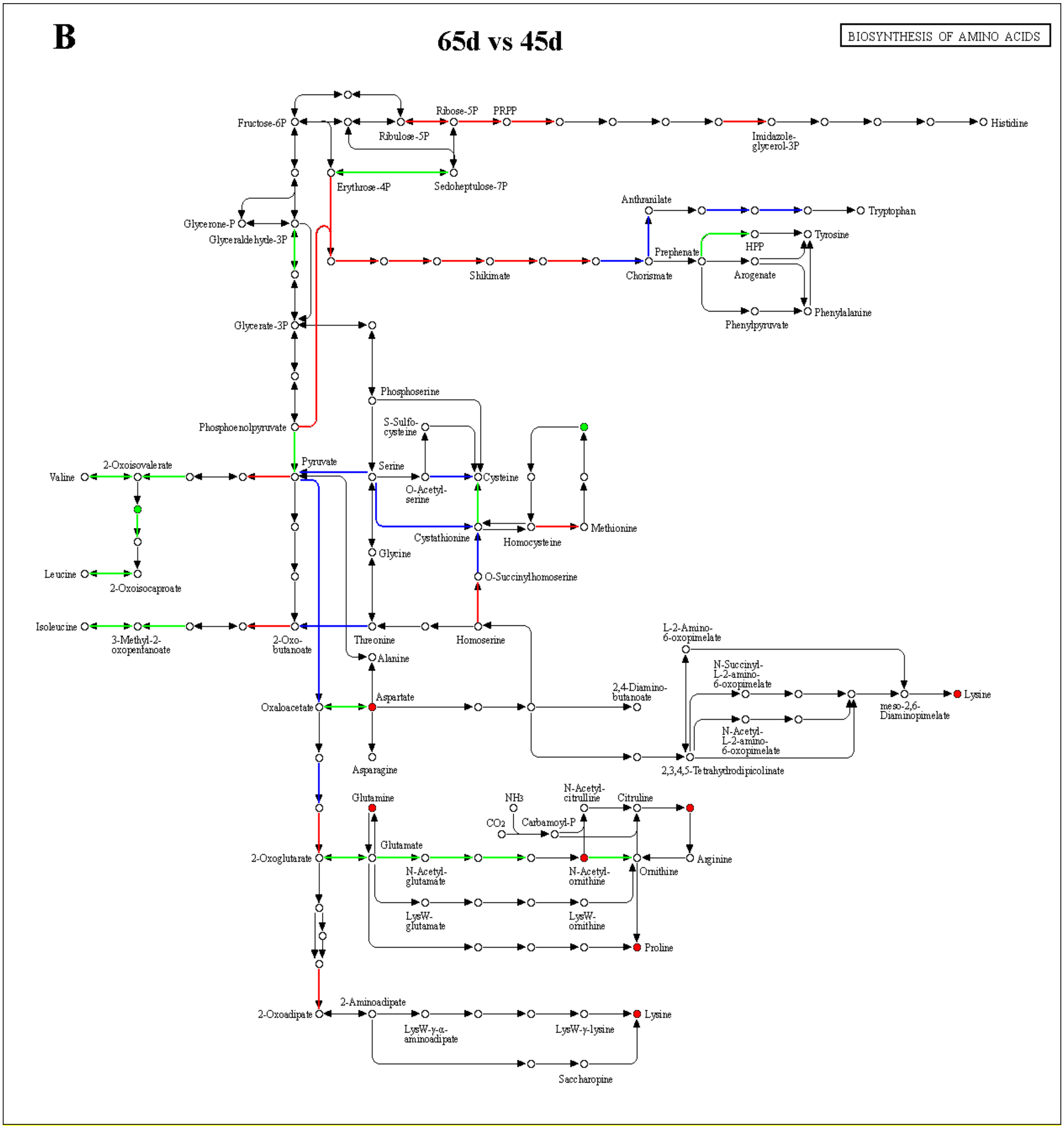
B
65d vs 45d
BIOSYNTHESIS OF AMINO ACIDS
Fructose-6P
Ribose-5P
PRPP
Ribulose-5P
Imidazole-glycerol-3P
Histidine
Erythrose-4P
Sedoheptulose-7P
Anthranilate
Tryptophan
Glycerone-P
Glyceraldehyde-3P
Tyrosine
Prephenate
HPP
Shikimate
Chorismate
Arogenate
Glycerate-3P
Phenylalanine
Phenylpyruvate
Phosphoserine
Phosphoenolpyruvate
S-Sulfo-cysteine
2-Oxoisovalerate
Pyruvate
Serine
Valine
Cysteine
O-Acetyl-serine
Methionine
Cystathionine
Homocysteine
Glycine
Leucine
O-Succinylhomoserine
2-Oxoisocaproate
Isoleucine
3-Methyl-2-oxopentanoate
2-Oxo-butanoate
Threonine
Homoserine
L-2-Amino-6-oxopimelate
Alanine
N-Succinyl-L-2-amino-6-oxopimelate
2,4-Diamino-butanoate
Aspartate
Lysine
Oxaloacetate
meso-2,6-Diaminopimelate
N-Acetyl-L-2-amino-6-oxopimelate
Asparagine
2,3,4,5-Tetrahydrodipicolinate
N-Acetyl-citrulline
Glutamine
NH3
Citrulline
CO2
Carbamoyl-P
Glutamate
2-Oxoglutarate
Arginine
N-Acetyl-glutamate
N-Acetyl-ornithine
Ornithine
LysW-glutamate
LysW-ornithine
Proline
2-Aminoadipate
2-Oxoadipate
Lysine
LysW-γ-α-aminoadipate
LysW-γ-lysine
Saccharopine

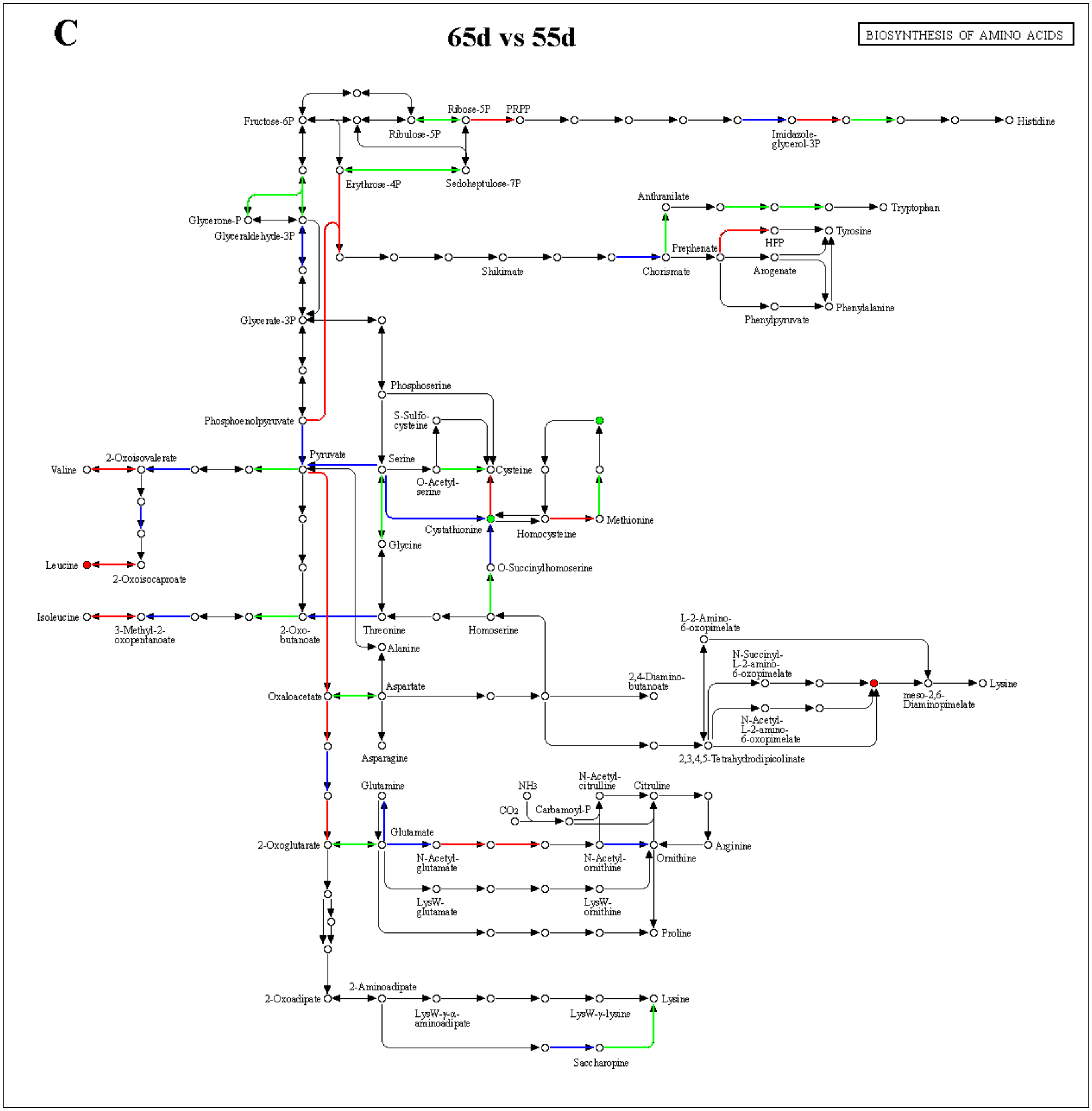

Figure S1. Biosynthesis of amino acids.

The circles and arrows represent metabolites and genes, respectively; red: up-regulated, blue: both up-regulated and down-regulated; green: down-regulated.
